# Toileting difficulties in older people with and without dementia receiving formal in‐home care—A longitudinal study

**DOI:** 10.1002/nop2.289

**Published:** 2019-05-17

**Authors:** Frida Grimsland, Arnfinn Seim, Tom Borza, Anne‐Sofie Helvik

**Affiliations:** ^1^ Department of Public Health and Nursing, Faculty of Medicine and Health Sciences Norwegian University of Science and Technology (NTNU) Trondheim Norway; ^2^ Centre for Old Age Psychiatric Research Innlandet Hospital Trust Ottestad Norway; ^3^ General Practice Research Unit, Department of Public Health and Nursing, Faculty of Medicine and Health Sciences Norwegian University of Science and Technology (NTNU) Trondheim Norway; ^4^ St Olavs University Hospital Trondheim Norway; ^5^ Norwegian National Advisory Unit on Ageing and Health Vestfold Health Trust Tønsberg Norway

**Keywords:** cognitive impairment, elderly, home care, incontinence, long‐term care, transferral

## Abstract

**Aim:**

To estimate the prevalence of toileting difficulties over time among older people (≥70 years) with and without dementia receiving formal in‐home care at baseline and to explore whether dementia at baseline was associated with toileting difficulties at the last assessment when adjusting for relevant covariates. We hypothesize that those with dementia have a higher prevalence and that baseline dementia is associated with toileting difficulties at last follow‐up.

**Design:**

A longitudinal observational study with three assessments over 36 months. Older people (≥70 years) from 19 Norwegian municipalities with in‐home care needs were included. The participants and their next of kin were interviewed.

**Method:**

In total, 1,001 (68% women) persons with a mean (*SD*) age 83.4 (5.7) years participated at baseline. Toileting difficulties were assessed using Lawton and Brody's Physical Self‐Maintenance Scale and Individual Nursing and Care Statistics. Information on physical comorbidity, number of prescribed drugs, cognitive function and formal care given was included. Dementia was diagnosed based on all information gathered.

**Results:**

At all time points, toileting difficulties were more prevalent in people with than without dementia. In adjusted analyses, dementia at baseline was associated with toileting difficulties at the last assessment. Nursing home admission was associated with increased odds for toileting difficulties.

## 1 INTRODUCTION

In the years to come, due to an ageing population and long life expectancy, an increase in the proportion of older people is expected (Syse, Pham, & Keilman, 2016). In Norway, recent predictions state that by 2060, approximately 20% will be aged 70 or older in a population with 7 million people (Syse et al., 2016). Increasing age is associated with reduced function and increased morbidity (Marengoni et al., 2011), and toileting difficulties are one of the challenges experienced (Buckley, Lapitan, & Epidemiology Committee of the Fourth International Consultation on Incontinence, 2010; Sharma, Yuan, Marshall, Merrie, & Bissett, 2016). Toileting difficulties are known to be challenging on a personal and economical level for both the individuals themselves and their families, to add work load on formal caregivers and to burden the society in general (Finne‐Soveri, Sorbye, Jonsson, Carpenter, & Bernabei, 2008; Milsom et al., 2014; Miner, 2004).

In Scandinavia, the public health and social care are legally obligated to provide public services to all inhabitants (Helse‐og Omsorgsdepartementet, 2011) and the formal care they provide contribute to letting older people with care needs stay in their homes for a longer period of time than what would be possible without such care (Otnes, 2015). In 2014, 15.3% of older people (≥66 years) in Norway received some kind of formal in‐home care and the total cost for such care was about 50 billion NOK (5.74 billion USD) (Abrahamsen, 2016; Førland & Folkestad, 2016).

Information about morbidity, functional status and need of care in older people is essential to politicians and healthcare planners responsible for providing adequate care to a reasonable cost. Previous studies with older people receiving formal in‐home care have focused on prevalence of dementia and neuropsychiatric symptoms and use of psychotropic drugs, as well as admission to nursing home and mortality (Abrahamsen, 2016; Wergeland, Selbaek, Bergh, Soederhamn, & Kirkevold, 2015; Wergeland, Selbaek, Hogset, Soderhamn, & Kirkevold, 2014). All the above‐mentioned factors influence type and amount of care delivered to community‐dwelling older people. In this regard, another factor of importance is toileting difficulties. Consequences of inadequate care and treatment of toileting difficulties may contribute to increasing functional decline, skin infections, risk of pressure ulcers, depression, loss of social interaction and social isolation, impairment of quality of life and also negative consequences for the caregiver (Farage, Miller, Berardesca, & Maibach, 2007, 2008; Gotoh et al., 2009; Miner, 2004). It has also been found to be a risk factor for nursing home admission (Luppa et al., 2010). Some studies have indicated that toileting difficulties can contribute to nursing home admission (Thom, Haan, & Eeden, 1997; Thomas et al., 2004), but the methodological quality of such studies has been criticized, and the importance of toileting difficulties for nursing home admission is still under discussion (Holroyd‐Leduc, Mehta, & Covinsky, 2004).

### 1.1 Background

Studies exploring toileting difficulties in older people define and classify the phenomenon in various ways. The terms are somewhat, but not completely, overlapping (Buckley et al., 2010), and studies may use the same term but define it differently. In addition, methodological differences and differences in study samples may cause difficulties when comparing studies. Toileting difficulties are in our study defined as having urine and/or faecal incontinence and/or having difficulties with or requiring assistance with toileting. This way, our definition includes several different challenges, including reduced physical functioning and dementia‐related difficulties such as reaching, finding and recognizing the toilet (Yap & Tan, 2006). Since few studies define toileting difficulties to also include the need for assistance, knowledge on prevalence of toileting difficulties, according to our definition, has so far been scarce (Buckley et al., 2010). Some studies including community‐dwelling older US people are available, and prevalence of toileting difficulties defined as need of assistance related to toileting have been reported to be 6% (Lee, Lindquist, Segal, & Covinsky, 2006) and increasing to 45% among frail community‐dwelling older people who met criteria for nursing home admission (Carey et al., 2008).

Even so, several studies have assessed urinary and/or faecal incontinence (Buckley et al., 2010; Ng, Sivakumaran, Nassar, & Gladman, 2015). One European study of community‐dwelling older people of both genders receiving formal in‐home care reported the prevalence of urinary incontinence to be 46% (Du Moulin, Hamers, Ambergen, Janssen, & Halfens, 2008), while it is reported to be 37% among community‐dwelling older people of both genders receiving formal in‐home care in Norway (Sorbye et al., 2009). In Scandinavian community‐dwelling older women, the overall prevalence of urinary incontinence was reported to be 30% and increasing with age (Ebbesen, Hunskaar, Rortveit, & Hannestad, 2013) and this finding is comparable to what is found elsewhere (Buckley et al., 2010). In a small study on Norwegian community‐dwelling older men (74–75 years), the reported prevalence of urinary incontinence was 50% (Spigset et al., 1989) and higher than found elsewhere (ranging from 11%–34% in men) (Buckley et al., 2010). The prevalence of faecal incontinence in a sample of Scandinavian community‐dwelling older women is reported to be 5% in the lower age strata and increasing with age (Rommen, Schei, Rydning, H Sultan, & Morkved, 2012), which is comparable with other studies (Roberts et al., 1999; Teunissen, Bosch, Hoogen, & Lagro‐Janssen, 2004; Wu, Matthews, Vaughan, & Markland, 2015). In an international study on faecal incontinence in community‐dwelling older men, the prevalence was reported to be 11% (Roberts et al., 1999).

Internationally, the high prevalence of incontinence among people with dementia living in nursing homes is well documented (Milsom et al., 2009). Some studies report a higher occurrence of incontinence among community‐dwelling older people with dementia compared to those without dementia (Bauer, Schwarzkopf, Graessel, & Holle, 2014; Grant, Drennan, Rait, Petersen, & Iliffe, 2013; Hellstrom, Ekelund, Milsom, & Skoog, 1994). Also, the rate of diagnosis of urinary and faecal incontinence among community‐dwelling older people with dementia has been found to be approximately three and four times, respectively, higher than the rate of these diagnoses among community‐dwelling older people without dementia (Grant et al., 2013). Even so, since studies are few and their findings vary, the prevalence of incontinence among community‐dwelling older people with dementia is considered not to be established (Drennan, Rait, Cole, Grant, & Iliffe, 2013). Moreover, in the perspective on need of formal in‐home care in those with and without dementia, it is of importance to study the prevalence of toileting difficulties using a definition including need for assistance. More severe dementia is associated with reduced mobility, impaired ability to recognize and/or to transfer to the toilet and difficulties dressing, as well as to reduced cognitive ability to interpret and respond to the sensation of a full bladder or to rectal contractions (Jirovec & Wells, 1990; Potter & Wagg, 2005).

Toileting difficulties including incontinence are still considered taboo (Day, Patricia, Loughran, & O'Sullivan, 2014; Specht, 2005). Lack of knowledge in the community in general may contribute to the suffering associated with such difficulties (Specht, 2005). Several studies have found that toileting difficulties including incontinence have a major impact on quality of life (Stenzelius, Mattiasson, Hallberg, & Westergren, 2004; Teunissen et al., 2004), mental health and social participation (Bedretdinova, Fritel, Zins, & Ringa, 2016; Felde, Bjelland, & Hunskaar, 2012; Stenzelius et al., 2004). Especially when combined with dementia, incontinence may cause great stress for both the individual and its caregivers (Finne‐Soveri et al., 2008; Gove et al., 2016; Grant et al., 2013; Thomas et al., 2004). In a review recently published, great concern was expressed about the lacking knowledge on toileting difficulties in community‐dwelling older European people with dementia (Gove et al., 2016). Among community‐dwelling older people in Norway receiving formal in‐home care, the prevalence of dementia is reported to be 42% (Wergeland et al., 2014), but information about the prevalence of toileting difficulties and whether the prevalence of toileting difficulties is higher in this group compared to those without dementia has been lacking.

Knowledge on the negative impact of toileting difficulties on the life of the individual and its surroundings and on how devastating such difficulties can be when combined with dementia motivated the conduction of this study. Our aim was to estimate the prevalence of toileting difficulties over time among people with and without dementia aged ≥70 years receiving formal in‐home care at the time of study inclusion (baseline). Moreover, we wanted to explore whether dementia at baseline was associated with toileting difficulties at the last assessment when adjusting for relevant covariates such as socio‐demographic factors, physical health, type of support at baseline and nursing home admission at a later stage. Firstly, we hypothesized that older people with dementia have a higher prevalence and that dementia at baseline is associated with toileting difficulties at last follow‐up. Secondly, we hypothesized that there is an association between nursing home admission after baseline and toileting difficulties at last follow‐up.

## 2 DESIGN

This is a longitudinal observational study based on three assessments (T1‐3) conducted with 18 months between each assessment.

## 3 METHOD

### 3.1 Participants

The participants were recruited from 19 municipalities in five counties in the eastern part of Norway. Both urban and rural municipalities of various sizes were included. Participants were aged ≥70 years and received formal in‐home care. Established users were recruited from registers on formal in‐home care, and new users were included successively. A next of kin responded on behalf of the participants on questions about physical self‐maintenance including toileting difficulties. Therefore, regardless of the kind and amount of services received, only people with a next of kin who saw them at least once a week were potential participants for selection.

**Figure 1:**
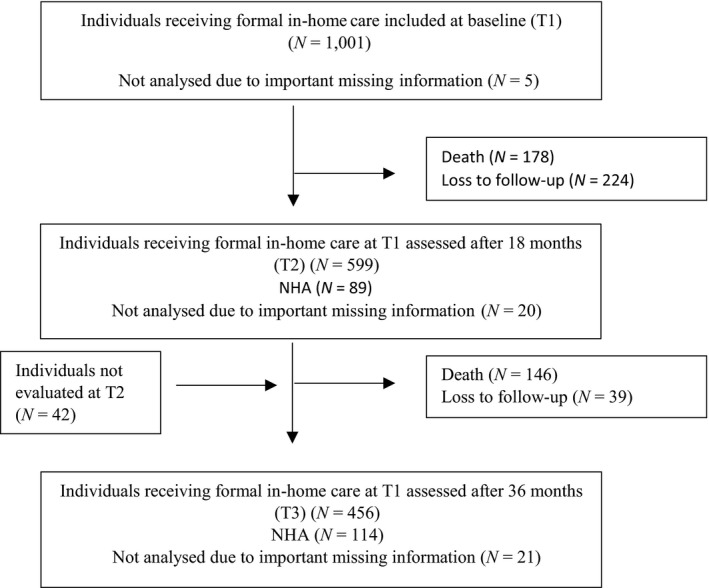
Flow chart of attrition from baseline (T1) to the last assessment (T3). Important missing information, Lacking information on both items assessing toileting difficulties. NHA, Nursing home admission

Of 1,796 eligible people, 795 declined to participate (Wergeland et al., 2014). In total, 1,001 older people (≥70 years) receiving formal in‐home care were included in the study between August 2008–December 2010 (Figure 1). The mean age (*SD*) of those who declined were higher, that is 85.0 (6.2) years versus mean age 83.4 (5.7) years (*p* < 0.001). Those who declined were also more often women than men (73.0% vs. 68.1%, *p* = 0.004) compared to those who were included in the study (Wergeland et al., 2014).

In total, 996 people met the criteria for inclusion in the analyses at baseline (T1). At T1, 5 persons were not included in the analysis due to missing all information on toileting difficulties. At the last assessment (T3), 456 persons were included. In total, 21 persons were not included in the analysis at T3 due to missing all information on toileting difficulties.

## 4 MEASURES

*Toileting difficulties* were assessed using items from Lawton and Brody's Physical Self‐Maintenance Scale (PSMS) (Lawton & Brody, 1969) and Individual Nursing and Care Statistics (IPLOS) (Helse‐og Omsorgsdepartementet, 2006) (see Table 1). PSMS has frequently been used in Norwegian and Scandinavian studies (Helvik, Skancke, & Selbaek, 2010; Selbaek, Kirkevold, & Engedal, 2007), and IPLOS is a national, pseudonymous register for individualized health and social care, created to gather and process information about the functional level and need of assistance for people receiving such care in the Norwegian municipalities (Helse‐og Omsorgsdepartementet, 2006).

**Table 1:** Response options of two items assessing toileting difficulties and how they were categorized for analytic purposes

Response options	Categorizations for analytic purposes	
**Toileting difficulties in PSMS**		
1 Care for self at toilet completely: no incontinence	1 Care for self at toilet completely: no toileting difficulties	1 No toileting difficulties
2 Needs to be reminded, or needs help in cleaning self, or has rare (weekly at most) accidents	2 Needs to be reminded, or needs help in cleaning self, or has rare (weekly at most) accidents	2 Has toileting difficulties
3 Soiling or wetting while asleep more than once a week	3 Soiling or wetting while asleep or awake more than once a week	
4 Soiling or wetting while awake more than once a week		
5 No control of bowels or bladder	4 No control of bowels or bladder	
**Toileting difficulties in IPLOS**		
1 No problems	1 No toileting difficulties	1 No toileting difficulties
2 Problems not causing need of assistance		
3 Some need of assistance	2 Some need of assistance	2 Has toileting difficulties
4 Extensive need of assistance	3 Extensive and full need of assistance	
5 Full need of assistance		

Note

Abbreviation(s): IPLOS, Individual Nursing and Care Statistics; PSMS, Physical Self‐Maintenance Scale.

One of six items in PSMS covers toileting functioning. The next of kin answered the measure on behalf of the participant. The item has five response options ranging from “no incontinence or need of assistance” (1) to “no control of bladder or bowel” (5). For analytic purposes, firstly the response categories 3 and 4 were merged, and secondly, the response categories 2–5 were merged.

The item in the IPLOS form concerning toileting was reported by health personnel and had five response options: “No problems” (1), “Problems not causing need of assistance” (2), “Some need of assistance” (3), “Extensive need of assistance” (4) and “Full need of assistance” (5). For analytic purposes, firstly the response categories 1 and 2 as well as categories 4 and 5 were merged, and secondly, the categories 1 and 2 as well as categories 3–5 were merged.

*Cognitive functioning* was evaluated using Mini‐Mental State Examination (MMSE) (Folstein, Folstein, & McHugh, 1975), Clock Drawing Test (CDT) (Shulman, 2000) and Informant Questionnaire on Cognitive Decline in the Elderly (IQ‐CODE) (Jorm, 2004). The MMSE consisted of 30 point giving tasks administered by an interviewer. A higher score indicated a better cognitive functioning (Folstein et al., 1975). The result of the CDT was rated from 1–5, and 5 was a “perfect clock” (Shulman, 2000). The IQ‐CODE assessed changes in cognitive function over the past 10 years by interviews with the closest proxy. The score 3 meant “no change,” while values >3 indicated loss of function and a score <3 indicated improvement (Jorm, 2004).

*The severity of dementia* was described by the use of Clinical Dementia Rating Scale (CDR) (Hughes, Berg, Danziger, Coben, & Martin, 1982). The CDR was divided into six parts and investigated memory, orientation, judgement and problem‐solving, community affairs, home and hobbies and personal care. A total score of 0 meant “no dementia,” while a score of 3 was given when the dementia was severe. The scores in between were 0.5 (possible), 1 (mild) and 2 (moderate) (Hughes et al., 1982).

*Diagnosis of dementia*: Two experienced physicians in clinical geriatric psychiatry and research independently diagnosed dementia according to the ICD‐10 criteria based on all the information gathered from the participants and their next of kin. A third clinical expert was consulted when disagreement between the two main clinicians occurred, and a consensus was reached.

*Physical comorbidity* was assessed using the General Medical Health Rating (GMHR) scale with a four‐point response score (Lyketsos et al., 1999). The number and severity of general physical conditions and the use of drugs due to such conditions are considered prior to scoring from very good (1) to poor (4) (Lyketsos et al., 1999). For analytic purposes, the GMHR responses were dichotomized to poor versus not poor including the response options: fairly good, good and very good.

*Number of drugs prescribed* was registered using the medical record of each patient.

*Demographic information* was collected as a part of the general baseline examination and included marital status, age and gender.

*Type of formal care* received was registered. At T1, receiving formal in‐home care included domiciliary care, in‐home nursing care and “other types of support” (alternative answers were yes or no). At T2 and T3, also nursing home admission was registered.

### 4.1 Procedure

A research nurse coordinated the collection of data. The participants and their next of kin were interviewed in their own homes. The interviews were done simultaneously by two separate assessors. The interviewers were mostly nurses, social educators and occupational therapists. Before collecting the baseline data, the interviewers participated in a 2‐day course to learn how to use the assessment tools. Before subsequent assessments, interviewers participated in 1‐day courses.

### 4.2 Analysis

Data were analysed using IBM SPSS statistics Windows version 24. (IBM Corp.). To describe the data sample, chi‐squared statistics for categorical data and two sample *t* test (two‐tailed) (due to normality of distribution) for continuous data were used. *p*‐Values below 0.05 were considered statistically significant. All tests were two‐sided.

In participants at T1‐3, toileting difficulties measured with PSMS and IPLOS by dementia was compared using chi‐squared test. Bivariate logistic regression was used to explore factors associated with toileting difficulties measured with PSMS and IPLOS (yes/no) at T3. Independent variables were socio‐demographics (age, gender and marital status), health condition (GMHR, prevalence of dementia and number of drugs), type of support given and toileting difficulties at baseline as well as nursing home admission at a later time point. We checked for significant interaction between age and health condition variables and the outcomes. If association between toileting difficulties and a measure had a *p*‐Value of ≤0.250 in the unadjusted analysis for one of the two outcomes, the measure was included in the adjusted analysis. This was done to avoid omitting variables with a potentially important influence on the outcomes. The associations were presented using odds ratio (OR) and 95% confidential intervals (95% CI).

### 4.3 Ethical considerations

Before participation, both participants and their next of kin received oral and written information about the project. Written consents were obtained from participants and their next of kin. When participants were unable to give consent, their closest family proxy gave informed consent on behalf of them. The project was approved by the Directorate for Health and Social Affairs (08/2984), the Norwegian Social Science Data Services (NSD) (07–2008SI) and the Regional Committee for Medical and Health Research Ethics for Eastern Norway (S‐08111b).

## 5 RESULTS

### 5.1 Sample characteristics

Of the 996 baseline (T1) participants included in the analysis, the mean age (*SD*) was 84.3 (5.6) years and 679 (68.2%) participants were women (see Table 2). Dementia was diagnosed in 412 participants (41.4%) at T1. In total, 579 (58.1%) participated in the analysis at T2 and 435 (43.7%) at T3 (see Figure 1). In total, 324 participants died during the follow‐up period. At T2, 89 (14.4%) persons were nursing home residents, and at T3, 114 (25.2%) persons were nursing home residents.

**Table 2:** Baseline characteristics of participants by dementia/no dementia

	Total	D	nD	*p*‐Values^a^
Participants *N* (%)	996	412(41.4)	584 (58.6)	
Socio‐demographics				
Age, Mean (*SD*)	83.4 (5.7)	84.6 (5.5)	82.5 (5.6)	≤0.001
Female, *N* (%)	679 (68.2)	271 (27.2)	408 (41.0)	0.173
Living with partner/married^a^, *N* (%)	296 (29.7)	131 (13.2)	165 (16.6)	0.235
Health condition				
GMHR^b^poor (vs. fairly good/good/very good), *N* (%)	105 (10.6)	57 (5.7)	48 (4.8)	0.004
MMSE, Mean (*SD*)	24.5(4.8)	20.1 (4.1)	27.6 (2.0)	≤0.001
Number of drugs, Mean (*SD*)	5.3(2.9)	5.4 (2.9)	5.3 (2.9)	0.688
Type of in‐home care				
Domiciliary care^c^, *N* (%)	525 (53.1)	208 (21.0)	317 (32.1)	0.212
Nursing care^c^, *N* (%)	666 (67.3)	345 (34.9)	321(32.5)	≤0.001
Other types of support^c^, *N* (%)	546 (55.2)	249 (25.2)	297 (30.0)	0.003

Note

Abbreviation(s): D/nD, Dementia/no Dementia; GMHR, General Medical Health Rating; MMSE, Mini‐Mental State Examination.

a Missing values (*N* = 1).

b Missing values (*N* = 2).

c Missing values (*N* = 7).

Those included at T3 were more often women (*p* < 0.01), and their mean age (*SD*) at T1 was lower compared with the group who was lost to follow‐up or died during the time of follow‐up, that is 82.6 (5.4) years versus 84.1 (5.8) years, *p* < 0.01. The mean follow‐up time of those included at T3 was 36.3 (2.2) months.

### 5.2 Toileting difficulties at three time points

The prevalence of toileting difficulties among participants at T1‐3 is presented in Table 3. Throughout the study, independent of the measure used, and most participants had no difficulties or need of assistance with toileting. Over time, independent of the measure used, there was a change in the distribution of participants with an increase in toileting difficulties. At all time points, participants with dementia had more often toileting difficulties compared to those without dementia.

**Table 3:** Comparing toileting difficulties by dementia/no dementia at the assessment time points T1‐3

	T1			T2			T3		
	Total	D	nD	Total	D	nD	Total	D	nD
**PSMS**									
Total a, *N* (%)	996 (100.0)	412 (100.0)	584 (100.0)	576 (100.0)	294 (100.0)	282 (100.0)	433 (100.0)	224 (100.0)	209 (100.0)
1 No toileting difficulties, *N* (%)	886 (89.0)	325 (78.9)	561 (96.1)	470 (81.6)	199 (67.7)	271 (96.1)	321 (74.1)	123 (54.9)	198 (94.7)
2 Needs to be reminded, or needs help in cleaning self, or has rare (weekly at most) accidents, *N* (%)	33 (3.3)	28 (6.8)	5 (0.9)	44 (7.6)	39 (13.3)	5 (1.8)	45 (10.4)	40 (17.9)	5 (2.4)
3 Soiling or wetting while asleep/awake more than once a week, *N* (%)	64 (6.4)	49 (11.9)	15 (2.6)	49 (8.5)	45 (15.3)	4 (1.4)	47 (10.9)	42 (18.8)	5 (2.4)
4 No control of bowels or bladder, *N* (%)	13 (1.3)	10 (2.4)	3 (0.5)	13 (2.3)	11 (3.7)	2 (0.7)	20 (4.6)	19 (8.5)	1 (0.5)
*p*‐Value			≤0.001			≤0.001			≤0.001
**IPLOS**									
Total b, *N* (%)	985 (100.0)	406 (100.0)	579 (100.0)	565 (100.0)	282 (100.0)	283 (100.0)	423 (100.0)	215 (100.0)	208 (100.0)
1 No toileting difficulties, *N* (%)	902 (91.6)	344 (84.7)	558 (96.4)	470 (83.2)	203 (72.0)	267 (94.3)	310 (73.3)	118 (54.9)	192 (92.3)
2 Some need of assistance, *N* (%)	54 (5.5)	36 (8.9)	18 (3.1)	51 (9.0)	39 (13.8)	12 (4.2)	47 (11.1)	37 (17.2)	10 (4.8)
3 Extensive or full need of assistance, *N* (%)	29 (2.9)	26 (6.4)	3 (0.5)	44 (7.8)	40 (14.2)	4 (1.4)	66 (15.6)	60 (27.9)	6 (2.9)
*p*‐Value			≤0.001			≤0.001			≤0.001

Note

T1 Assessment at study inclusion, baseline; T2 Assessment 18 months after baseline; T3 Assessment 36 month after baseline.

Abbreviation(s): D/nD, Dementia/no dementia; PSMS, Physical Self‐Maintenance Scale; IPLOS, Individual Nursing and Care Statistics.

a Missing information on PSMS toileting difficulties at T2 (*N* = 3) and T3 (*N* = 2).

b Missing information on IPLOS toileting difficulties at T1 (*N* = 11), T2 (*N* = 14) and T3 (*N* = 12).

### 5.3 Factors associated with toileting difficulties at T3

In bivariate logistic analyses, we explored the possible association between socio‐demographics (age, gender and marital status), health condition (GMHR, prevalence of dementia and number of drugs), type of support given, toileting difficulties at baseline and nursing home admission at a later time point by toileting difficulties at T3 (see Table 4). In the adjusted analyses, independent of the measure used to assess toileting difficulties, dementia at baseline and nursing home admission after baseline were associated with toileting difficulties at T3. Baseline dementia increased the odds for toileting difficulties more than twofold, that is OR (95% CI) was 2.69 (1.47; 4.92) and 2.07 (1.14; 3.76) using PSMS and IPLOS outcome measures, respectively. Nursing home admission increased the odds for toileting difficulties more than ten times, that is OR was 11.12 (6.07; 20.38) and 10.60 (5.75; 19.53) using PSMS and IPLOS, respectively.

**Table 4:** Unadjusted and adjusted analysis of toileting difficulties (vs. no difficulties) measured with PSMS (*N* = 433) and IPLOS (*N* = 423) at T3^a^

	PSMS				IPLOS			
	Unadjusted OR (95% CI)	*p*‐Value	Adjusted OR (95% CI)	*p*‐Value	Unadjusted OR (95% CI)	*p*‐Value	Adjusted OR (95% CI)	*p*‐Value
**Characteristics at T1**								
Socio‐demographics								
Age	1.04 (1.00; 1,09)	0.036	1.00 (0.94; 1.05)	0.877	1.03 (0.99; 1.08)	0.130	0.98 (0.93; 1.03)	0.436
Male (vs. female)	1.29 (0.80; 2.08)	0.291	1.05 (0.53; 2.06)	0.894	1.61 (1.00; 2.58)	0.049	1.43 (0.74; 2.76)	0.284
Living with partner/married	1.07 (0.65; 1.74)	0.796	0.92 (0.46; 1.85)	0.812	1.39 (0.86; 2.25)	0.184	1.25 (0.62; 2.49)	0.532
Health condition								
GMHR—poor (vs. fairly good/good/very good)	2.71 (1.28; 5.76)	0.009	2.11 (0.76; 5.88)	0.151	2.04 (0.94; 4.42)	0.070	1.11 (0.36; 3.45)	0.861
Dementia (vs. no dementia)	6.21 (3.89; 9.92)	≤0.001	2.69 (1.47; 4.92)	≤0.001	5.31 (3.35; 8.43)	≤0.001	2.07 (1.14; 3.76)	0.017
Number of drugs	1.01 (0.94; 1,09)	0.785			0.99 (0.92; 1.07)	0.783		
Type of in‐home support								
Nursing care	3.87 (2.31; 6.47)	≤0.001	1.68 (0.89; 3.18)	0.113	4.14 (2.47; 6.93)	≤0.001	1.66 (0.87; 3.15)	0.121
Home aid	1.52 (0.98, 2.35)	0.062	1.56 (0.86, 2.83)	0.139	1.32 (0.85; 2.03)	0.217	1.39 (0.79; 2.47)	0.258
Other types of support	1.37 (0.88; 2.13)	0.161	0.77 (0.43; 1.40)	0.399	1.93 (1.22; 3.05)	0.005	1.74 (0.97; 3.11)	0.064
Toileting difficulties								
PSMS toileting difficulties yes^b^	9.03 (3.87; 21.06)	≤0.001	6.54 (2.22; 19.26)	≤0.001				
IPLOS toileting difficulties yes^c^					9.10 (3.49; 23.75)	≤0.001	8.53 (2.78; 26.20)	≤0.001
**Characteristics at T2 and/or T3**								
NHA	17.09 (10.09; 28.92)	≤0.001	11.12 (6.07; 20.38)	≤0.001	14.91 (8.83; 25.17)	≤0.001	10.60 (5.75; 19.53)	≤0.001

Note

Included independent variables in the adjusted analysis were those variables that had a *p*‐value < 0.250 in one of the two outcome variables describing toileting difficulties in the unadjusted analysis.

T1 Assessment at study inclusion, baseline; T2 Assessment 18 months after baseline; T3 Assessment 36 month after baseline.

Abbreviation(s): GMHR, General Medical Health Rating; IPLOS, Individual Nursing and Care Statistics; NHA, Nursing home admission; PSMS, Physical Self‐Maintenance Scale.

a Missing information at T3 on PSMS toileting difficulties (*N* = 2) and IPLOS toileting difficulties (*N* = 12) among those reporting toileting difficulties at least at one of the measures.

b PSMS toilet difficulties yes: From some need of assistance to no control of bowels and/or bladder. Reference level: No toileting difficulties.

c IPLOS toilet difficulties yes: From some need of assistance to full need of assistance. Reference level: No toileting difficulties.

## 6 DISCUSSION

This study explored the prevalence of toileting difficulties over time among older people who, at least at baseline, were community‐dwelling and received formal in‐home care. There was a perceptible increase in the prevalence of toileting difficulties among the participants throughout the follow‐up period. At all time points, groups with dementia had a higher prevalence of toileting difficulties than groups without dementia. In the adjusted analysis, we found that dementia and toileting difficulties at T1 and nursing home admission after T1 were associated with toileting difficulties at T3.

At baseline, about 10% of all the 996 participants had toileting difficulties. However, at T3, the last follow‐up 36 months later, about 25% of the remaining participants had toileting difficulties. Two different accepted measures (PSMS and IPLOS) assessing toileting difficulties gave similar results. The few previous studies addressing toileting difficulties defined as need of toileting assistance have reported a prevalence showing a similar pattern, from 6% among community‐dwelling US older people (Lee et al., 2006) to 45% among frail community‐dwelling older people who met criteria for nursing home admission (Carey et al., 2008).

International studies and studies in Scandinavia have reported the prevalence of urinary incontinence in similar groups to be equally high or higher (Buckley et al., 2010; Ebbesen et al., 2013; Milsom et al., 2014; Sorbye et al., 2009; Spigset et al., 1989) and faecal incontinence to be considerably lower (Finne‐Soveri et al., 2008; Ng et al., 2015; Roberts et al., 1999; Rommen et al., 2012; Wu et al., 2015) than the prevalence of toileting difficulties reported in our study. Our study provides information on challenges experienced and also on the need for help caused by these challenges, but we do not have information on whether assisting needs are linked specifically to toilet visits, urinary incontinence and/or faecal incontinence. The finding of an increasing prevalence of toileting difficulties over time, even if concepts are overlapping and not directly comparable, is consistent with results from previous longitudinal studies on incontinence among older people (Buckley et al., 2010; Ebbesen et al., 2013; Markland et al., 2010).

Independent of the measure used, we found the prevalence of toileting difficulties to be higher in the subgroup with dementia compared to the subgroup without dementia. This finding is consistent with previous studies which have reported high prevalence of toileting difficulties including incontinence in groups with dementia (Bauer et al., 2014; Grant et al., 2013; Hellstrom et al., 1994). In binary logistic analysis of toileting difficulties, dementia at baseline was found to be associated with toileting difficulties at T3, independent of the measure used and after adjusting for socio‐demographic and physical health differences at T1 as well as nursing home admission after T1. The syndrome of dementia can be caused by various underlying diseases and includes specific signs and symptoms of progressive deterioration of cognitive functions, in combination with a presumed underlying substrate of neuropathology (van der Flier & Scheltens, 2005). Previous studies have found that frailty in general and inclined ability to perform activities of daily living is linked to dementia (Helvik, Engedal, Benth, & Selbaek, 2014; Kojima, Taniguchi, Iliffe, & Walters, 2016). Toilet functioning depends on an interplay of several factors, such as cognitive and neurological function. Dementia may account for cognitive and functional deficits and possibly pathology in the urinary system and thereby affect the ability to stay continent and result in true or functional incontinence (Yap & Tan, 2006). Previous studies have stated that more severe dementia is associated with reduced mobility, impaired ability to recognize and/or to transfer to the toilet and difficulties dressing, as well as to reduced ability to cognitively interpret and respond to the sensation of a full bladder or to rectal contractions (Jirovec & Wells, 1990; Potter & Wagg, 2005). These studies support our findings in that those with dementia have a higher prevalence of toileting difficulties and that baseline dementia is associated with toileting difficulties at last follow‐up.

In the present study, we investigated the possible link between nursing home admission and toileting difficulties. Toileting difficulties were more prevalent in those experiencing nursing home admission after baseline compared to those receiving care at home throughout the study period. In adjusted analysis, the odds for toileting difficulties at T3 was tenfold higher in those with nursing home admission during the time of follow‐up, independent of the measure used. Our material does not allow us to make definite conclusions on whether toileting difficulties arose before and contributed to the nursing home admission or arose after the admission. Still, our results strongly suggest that there is an association between nursing home admission and toileting difficulties. It is reasonable to expect toileting difficulties to increase the likelihood of nursing home admission independent of the participants’ age, gender and dementia status. Previous research has also addressed the possible association between toileting difficulties and nursing home admission, and some studies have indicated that toileting difficulties can contribute to nursing home admission (Thom et al., 1997; Thomas et al., 2004). These results have been challenged by a review criticizing methodological quality of studies on a possible association between nursing home admission and toileting difficulties and by questioning the role of incontinence as an independent risk factor for nursing home admission (Holroyd‐Leduc et al., 2004; Luppa et al., 2010). A recent study investigated the role of urinary incontinence and faecal incontinence separately and found urinary incontinence, but not faecal incontinence, to be an independent risk factor for nursing home admission (Schluter, Ward, Arnold, Scrase, & Jamieson, 2017). A Norwegian study on nursing home admission and death using the same study material as used in our study found that lower personal functioning in activities of daily living (ADL) was associated with a higher risk of nursing home admission. Toileting difficulties in particular were not investigated, but were part of the ADL evaluation (Wergeland et al., 2015). In line with these previous findings and our new results about toileting difficulties, prevention of ADL decline and toileting difficulties should be of concern for those providing in‐home care and also for healthcare planners. Toileting difficulties developed after admission to nursing home have also been previously addressed, and some studies have found that prevalence of toileting difficulties can rise in a few months to years after admission (Bliss, Gurvich, Eberly, & Harms, 2017; Boguth & Schenk, 2008; Ihnat et al., 2016; Saga, Vinsnes, Morkved, Norton, & Seim, 2013; Saxer, Halfens, de Bie, & Dassen, 2008). Concerning incontinence among residents established in nursing homes, prevalence is repeatedly reported to be high (Ihnat et al., 2016; Offermans, Du Moulin, Hamers, Dassen, & Halfens, 2009; Saga et al., 2013; Saga, Vinsnes, Morkved, Norton, & Seim, 2015; Saxer et al., 2008). The seemingly complex relation between toileting difficulties and nursing home residency has been widely investigated, and the value of mapping and combating incontinence associated with nursing home residency is still being emphasized (Bliss et al., 2017; Offermans et al., 2009; Saga et al., 2013, 2015).

### 6.1 Strengths and limitations

The present study has several strengths. A high number of residents were available at baseline, and the study had a longitudinal design and well known and widely used measuring instruments were used. When toileting difficulties were investigated, two different assessment tools with some differences in wording were used. In our opinion, the differences in wording provides complementary information. A high number of participants and use of highly relevant measures made it possible to adjust for a variety of factors in the logistic regression analysis. Precautions concerning the assessors who gathered the information were taken. All the selected assessors were familiar with observing and cooperating with community‐dwelling older people and their families. Prior to collection of data, all assessors participated in standardized courses lasting 1–2 days.

The study has limitations that need to be addressed. Firstly, the associations found in our study should be interpreted with caution since our design does not allow for inferences about causality. Secondly, many from the original population refused participation at baseline and remaining participants differed from this population in mean age and gender distribution (Wergeland et al., 2014). Therefore, caution should be exercised in generalizing the study result to all recipients of formal in‐home care. Furthermore, the participants were only included if they had formal in‐home care from the municipality, and thus, a generalization to community‐dwelling older people in general should be avoided. Thirdly, due to high age, poor health and deaths, some participants were lacking information at one or two interviews. About one‐third of the participants had died during the follow‐up. The varying number of observations per individual may generate imbalanced data, and the high number of dropouts may result in attrition bias. Fourthly, the participants health and the data collection method used, made extensive examination difficult. The use of PSMS and IPLOS as measures for toileting difficulties gave thorough information on need for assistance but did not provide precise information on what type of toileting difficulties that was experienced. Measures more directly addressing degree and type of assistance needed, and degree of urinary and faecal incontinence could have given us additional information, strengthened our study and made it easier to compare our results with results from previous studies on incontinence. Lastly, the Norwegian public healthcare system is the context in which the material is collected and evidence for generalizability to healthcare systems outside Scandinavia is limited.

Even with its limitations, the study raises the awareness on toileting difficulties including incontinence in old age and especially in those living at home with care needs. Gathering knowledge on toileting difficulties both in those with and without dementia should have clinical implications. The importance of health professionals addressing toileting difficulties is amplified by the fact that such difficulties are still considered taboo and that older people may resign and minimize their symptoms (Horrocks, Somerset, Stoddart, & Peters, 2004; Specht, 2005). Addressing toileting difficulties may improve the situation of older people struggling with toileting difficulties, improve the well‐being of their informal care givers and in addition, avoid or prolong time before home admission. Older people with dementia are known to have an impaired ability to take care of their own health. Due to the high prevalence of toileting difficulties in older people with dementia, there should be a special focus on such difficulties in this group.

## 7 CONCLUSION

In a longitudinal study with three assessments conducted over 36 months in a sample aged ≥70 years receiving formal in‐home care at baseline, we found that toileting difficulties increased over time. People with dementia at baseline and/or with nursing home admission during the time of follow‐up, were more likely to have toileting difficulties during the time of follow‐up than those without such problems and/or needs.

## CONFLICTS OF INTEREST

The authors declare having no conflict of interest.

## AUTHOR CONTRIBUTIONS

FG analysed most of the data and drafted the majority of this manuscript, and A‐SH was responsible for quality assurance of the study results and participated in the analysis and drafted parts of the manuscript. All authors participated in interpreting the study results, editing the manuscript, as well as reading and approving the final manuscript.

## ETHICAL APPROVAL

The project was approved by the Directorate for Health and Social Affairs (08/2984), the Norwegian Social Science Data Services (NSD) (07–2008SI) and the Regional Committee for Medical and Health Research Ethics for Eastern Norway (S‐08111b).
